# Supplementary material for: The impact of digital communication and data exchange on primary health service delivery in a small island developing state setting
Source: PLOS Digit Health. 2022 Oct 7;1(10):e0000109. doi: 10.1371/journal.pdig.0000109 (PMC9931309; doi:10.1371/journal.pdig.0000109)
Supplement: S1 File — (DOCX) [file pdig.0000109.s001.docx]

**Tuvalu VSAT impact study – semi-structured key informant interview data collection tool**

**Questions**

1. I’d like to start by asking you to describe the facility you work at, the staff you work with, and the community you serve?
2. I understand that the VSAT system was installed in 2020, can you tell me about this?
3. One of the objectives of the VSAT was to improve communication between staff on the outer islands and doctors, nurses and specialists at Princess Margaret Hospital. Have you seen any evidence of this having happened? Can you provide examples?
4. Could you now tell me about what impact VSAT-enabled communication with staff Princess Margaret Hospital staff has had on your clinical practice?
5. [If VSAT has not had (or had only minimal) impact on communication with staff at Princess Margaret Hospital, why do you think this is so?]
6. One of the many challenges associated with providing healthcare on outer islands is access to a reliable supply of medicines and other health consumables, such as dressings and syringes. Has there been any change in the way stock is managed since the introduction of VSAT? Could you provide some examples?
7. How has VSAT impacted the way you and your interact with the patient?
8. Are there any other ways that VSAT has impacted the way you interact with the patients, the public or the broader health system?
9. Next, I would like to talk about the experience with integrating VSAT into your clinic. Could you tell me how it went, and how staff have taken to the system?
10. Did the staff have any concerns with using new technology? If so, could you explain what the concerns were and if they were addressed, how?
11. Could anything have been done differently to make the integration of VSAT smoother or more successful?
12. Think to the future now, what do you see as the most important factors that ought to be in considered to ensure the VSAT system is used to its maximum potential?
13. What challenges are there that may inhibit sustainable adoption of VSAT in Tuvalu?
14. Is there anything we haven’t discussed that you think would be useful to the study?
