# Supplementary material for: The impact of digital communication and data exchange on primary health service delivery in a small island developing state setting
Source: PLOS Digit Health. 2022 Oct 7;1(10):e0000109. doi: 10.1371/journal.pdig.0000109 (PMC9931309; doi:10.1371/journal.pdig.0000109)
Supplement: Attachment — Submitted filename: Review.pdf [file pdig.0000109.s002.pdf]

--REVIEW-- 19.04.2022

Satellites have a wide range of applications, of which the education, medical, banking, oil, and government sectors (such as crisis management, as exemplified by Japan) can be pointed out. They are used to provide communication wherever access to traditional systems is impossible or difficult (mountainous areas, remote regions, island countries such as Tuvalu, etc.). With the help of satellites and VSAT systems, the cheapest global communication system can be applied. For this reason, the use of cheap and small VSAT antennae is so common in such locations, and often becomes the only possibility of communication. In practice, the VSAT systems are widely applied for filling so called “white spots” of access to the Internet or telephones (there are special government programs dedicated for this purpose, e.g. indicated by the Authors of the Tuvalu's digital health strategies).

Authors write “VSAT, while considered a suitable technology for Tuvalu, where land-based digital telecommunications infrastructure is lacking, is one of many ICT options available. Where land-based and mobile digital networks are present, authorities may find it more cost-effective to invest in the facility-based infrastructure and personal internet connectable devices required to access commercial telecommunications services”.

In recent years, such terminals have been used to access broadband Internet (e.g. from the HTS KA-SAT satellite). The implementation of CONECT VHTS may allow prices to decrease to those offered by fiber Internet providers in the long term. And this should be emphasized by the Authors.

The article presented by the Authors is valuable and in my opinion deserves to be published. As the medical staff of Tuvalu in 2021 included 13 general doctors and three specialists (an anesthesiologist, an obstetrician, and a gynecologist), certainly access to modern VSAT technologies will help to raise the standard of living of the inhabitants of such remote places, including through telemedicine, online consultations, remote surgeries, or access to many Internet-based services.

However, before publication, it would be useful to consider improving certain elements of the article. I have provided a list of them below.

1). The article has an unusual structure first there is an introduction and then immediately afterwards a results section. It would be worthwhile to consider correcting the content layout.

2). The research methodology (missing element) and limitations of the study should be described beforehand.

3). The comparison criteria should be better specified.

4). Summary should be at the end of the article.

5). It would be useful to consider adding detailed data on the satellite systems/solutions used in Tuvalu (the data currently presented is residual).

6). **The most important comment**! It would be helpful to illustrate the data in the form of pie charts (the missing element). As it is, presented descriptions without diagrams are difficult to read. In each of the subsections it would be worthwhile to supplement this. Also, there is a lack of exact percentage statistics in the five main themes considered. This should be corrected.

7). It would be useful to show reference to other reports on ICT use within the area (examples of methodology can be found in many works).

8). Pls complete the bibliography on the use of VSAT systems e.g.:

M. Sasanuma, H. Uchiyama, T. Nagoya, M. Furukawa, T. Motohisa, Research and development of very small aperture terminals (VSAT) that can be installed by easy operation during disasters – Issues and the solutions for implementing simple and easy installation of VSAT earth station, IEICE Tech. Rep., Vol. 112 (2013), pp. 1–3.

N. Suematsu, H. Oguma, S. Eguchi, S. Kameda, M. Sasanuma, K. Kuroda, Multi-mode SDR VSAT against big disasters, in: European Microwave Conference, EuMC, Nuremberg, 2013. doi: 10.23919/EuMC.2013.6686788.

S. Kameda, T. Okuguchi, S. Eguchi, N. Suematsu, Development of satellite-terrestrial multimode VSAT using software defined radio technology, in: Asia-Pacific Microwave Conference, IEEE, Sendai, 2014.

9). Authors write “VSAT remains vulnerable to broader system disruptions” – it would be worthwhile to justify it. There are many resources on the Internet. Examples of literature are here: https://doi.org/10.15244/pjoes/73907, https://doi.org/10.15244/pjoes/73906, https://doi.org/10.15244/pjoes/73906. It would be useful to complete the bibliography in this area as well.

10). There are minor editing errors in the article, such as a period in the wrong place after citations.

11). Figure 2 is directly taken from the article cited in the bibliography, i.e. Wilk-Jakubowski J (2021) A review on information systems engineering using vsat networks and their development directions. Yugosl J Oper Rres 31 (3): 409–428. doi:10.2298/YJOR200215015W. The Authors did not provide a source for the figure in the caption. If they have the last publisher's approval it should be sent to the publisher. Pls provide a footnote to this caption, i.e. [12] to avoid plagiarism.
