# Supplementary material for: The impact of digital communication and data exchange on primary health service delivery in a small island developing state setting
Source: PLOS Digit Health. 2022 Oct 7;1(10):e0000109. doi: 10.1371/journal.pdig.0000109 (PMC9931309; doi:10.1371/journal.pdig.0000109)
Supplement: Attachment — Submitted filename: R1_ Response to journal and reviewers comments_final.docx [file pdig.0000109.s003.docx]

**Response to journal and reviewers' comments (R1)**

**Manuscript title: The impact of digital communication and data exchange on primary health service delivery in a small island developing state setting**

**Submission number: PDIG-D-22-00057**

**Journal Requirements**

1. Please amend your detailed Financial Disclosure statement. This is published with the article. It must therefore be completed in full sentences and contain the exact wording you wish to be published. a. State the initials, alongside each funding source, of each author to receive each grant.

b. State what role the funders took in the study. If the funders had no role in your study, please state: "The funders had no role in study design, data collection and analysis, decision to publish, or preparation of the manuscript."

Response: The following text has been added. "This research was funded by a grant received by ATC from the Asia Pacific Observatory for Health Systems and Policies (Grant number 2020/1002187-0). The funders had no role in study design, data collection and analysis, decision to publish, or manuscript preparation."

2. Please update your online Competing Interests statement. If you have no competing interests to declare, please state: "The authors have declared that no competing interests exist."

Response: The text "The authors have declared that no competing interests exist" has been added.

3. Please provide separate figure files in .tif or .eps format and remove any figures embedded in your manuscript file. Please also ensure that all files are under our size limit of 10MB.

For more information about how to convert your figure files please see our guidelines: <https://journals.plos.org/digitalhealth/s/figures>

Response: Figures have been removed and uploaded in the format requested.

4. All figures and supporting information files will be published under the Creative Commons Attribution License (creativecommons.org/licenses/by/4.0/). Authors retain ownership of the copyright for their article and are responsible for third-party content used in the article.

Figure 1: please (a) provide a direct link to the base layer of the map used and ensure this is also included in the figure legend; (b) provide a link to the terms of use / license information for the base layer. We cannot publish proprietary or copyrighted maps (e.g. Google Maps, Mapquest) and the terms of use for your map base layer must be compatible with our CC-BY 4.0 license.

If your map was obtained from a copyrighted source please amend the figure so that the base map used is from an openly available source. Alternatively, please provide explicit written permission from the copyright holder granting you the right to publish the material under our CC-BY 4.0 license.

Please note that the following CC BY licenses are compatible with PLOS license: CC BY 4.0, CC BY 2.0 and CC BY 3.0, meanwhile such licenses as CC BY-ND 3.0 and others are not compatible due to additional restrictions.

If you are unsure whether you can use a map or not, please do reach out and we will be able to help you. The following websites are good examples of where you can source open access or public domain maps:

* U.S. Geological Survey (USGS) - All maps are in the public domain. (<http://www.usgs.gov>)

* PlaniGlobe - All maps are published under a Creative Commons license so please cite "PlaniGlobe, <http://www.planiglobe.com>, CC BY 2.0" in the image credit after the caption. (<http://www.planiglobe.com/?lang=enl>)

* Natural Earth - All maps are public domain. (<http://www.naturalearthdata.com/about/terms-of-use/>)

Please upload any written confirmation as an 'Other' file type. It must clarify that the copyright holder understands and agrees to the terms of the CC BY 4.0 license; general permission forms that do not specify permission to publish under the CC BY 4.0 will not be accepted. Note that uploading an email confirmation is acceptable.

Response: Copyright details for Figure 1 are now included in the caption. Figure 2 has been 'redrawn' with acknowledgement provided in the caption.

5. Please review your reference list to ensure that it is complete and correct. If you have cited papers that have been retracted, please include the rationale for doing so in the manuscript text, or remove these references and replace them with relevant current references. Any changes to the reference list should be mentioned in the rebuttal letter that accompanies your revised manuscript. If you need to cite a retracted article, indicate the article's retracted status in the References list and also include a citation and full reference for the retraction notice.

Response: Noted. The reference list has been reviewed.

**Reviewer #1:**

R1.1. Satellites have a wide range of applications, of which the education, medical, banking, oil, and government sectors (such as crisis management, as exemplified by Japan) can be pointed out. They are used to provide communication wherever access to traditional systems is impossible or difficult (mountainous areas, remote regions, island countries such as Tuvalu, etc.). With the help of satellites and VSAT systems, the cheapest global communication system can be applied. For this reason, the use of cheap and small VSAT antennae is so common in such locations, and often becomes the only possibility of communication. In practice, the VSAT systems are widely applied for filling so called "white spots" of access to the Internet or telephones (there are special government programs dedicated for this purpose, e.g. indicated by the Authors of the Tuvalu's digital health strategies).

Authors write "VSAT, while considered a suitable technology for Tuvalu, where land-based digital telecommunications infrastructure is lacking, is one of many ICT options available. Where land-based and mobile digital networks are present, authorities may find it more cost-effective to invest in the facility-based infrastructure and personal internet connectable devices required to access commercial telecommunications services".

In recent years, such terminals have been used to access broadband Internet (e.g. from the HTS KA-SAT satellite). The implementation of CONECT VHTS may allow prices to decrease to those offered by fiber Internet providers in the long term. And this should be emphasised by the Authors.

Response: The comments provided are interesting and useful, and we thank the reviewer for them. A sentence has been added at around line 284 acknowledging (without promoting a particular company) that satellite communication technology is rapidly evolving and that this may reduce costs.

R1.2. The article presented by the Authors is valuable and in my opinion deserves to be published. As the medical staff of Tuvalu in 2021 included 13 general doctors and three specialists (an anesthesiologist, an obstetrician, and a gynecologist), certainly access to modern VSAT technologies will help to raise the standard of living of the inhabitants of such remote places, including through telemedicine, online consultations, remote surgeries, or access to many Internet-based services.

However, before publication, it would be useful to consider improving certain elements of the article. I have provided a list of them below.

R1.2.1. The article has an unusual structure first there is an introduction and then immediately afterwards a results section. It would be worthwhile to consider correcting the content layout.

Response: The article is structured per the order outlined in the manuscript organisation section of the submission guidelines, which indicates that the methods are to be presented near the end of the manuscript.

R1.2.2. The research methodology (missing element) and limitations of the study should be described beforehand.

Response: The study's limitations are presented in the second last paragraph of the discussion section (around line 351). This, to our understanding, is standard practice and given the submission guidelines do not suggest limitations be discussed elsewhere, we have left the content as is.

R1.2.3. The comparison criteria should be better specified.

Response: The study is qualitative in design; hence, a comparison group or criteria are not required.

R1.2.4. Summary should be at the end of the article.

Response: The manuscript's structure is per the journal's submission guidelines, which place material and methods at the end of the article and does not require a 'conclusion' section/subheading. Given this, we have not included a conclusion subheading but rather summed up the key points of the research in the last paragraph (around line 363) of the discussion.

R1.2.5. It would be useful to consider adding detailed data on the satellite systems/solutions used in Tuvalu (the data currently presented is residual).

Response: We draw attention to the system description provided in the introduction section, at around line 104. We emphasise that the focus of the research is on the impact the introduction of VSAT has on service delivery in Tuvalu, and not on the system's design. Regardless, in an attempt to address this reviewer concern, we sought information for our Tuvaluan collaborator but, sadly, they are dealing with a significant life-threatening illness and have, understandably, not responded.

R1.2.6. The most important comment! It would be helpful to illustrate the data in the form of pie charts (the missing element). As it is, presented descriptions without diagrams are difficult to read. In each of the subsections it would be worthwhile to supplement this. Also, there is a lack of exact percentage statistics in the five main themes considered. This should be corrected.

Response: As this manuscript reports qualitative research, we purposefully avoided reporting the number/per cent of respondents who agreed/disagreed with certain statements; instead, we used the common language and quotes to highlight themes that emerged. Our reasoning for this is based on the work of multiple quantitative methodologists including Gilgun (2020), Blignault and Ritchie (2009) and Terry et al (2013), that suggest reporting of percentages have little meaning in almost all forms of qualitative research because the ‘purpose of the research is to identify patterns of meaning and not distributions of variables’ and that to report percentages ‘invites inappropriate comparisons and encourages quantitative rather than qualitative thinking.’ Given this, we have not added percentages in the or added tables/charts reporting proportions of responses.

Gilgun JF. Writing Up Qualitative Research. In: Leavy P, editor. The Oxford handbook of qualitative research. Second edition. New York, NY: Oxford University Press; 2020. p. 983–1011 (Oxford handbooks).

Blignault I, Ritchie J. Revealing the wood and the trees: reporting qualitative research. Health Promot J Austr 2009; 20(2):140–5. doi:10.1071/he09140.

Terry G, Hayfield N, Clarke V, Braun V. Thematic Analysis. In: Willig C, Stainton Rogers W, editors. The SAGE handbook of qualitative research in psychology. Second edition. Los Angeles: SAGE; 2017. p. 17–36.

R1.2.8. It would be useful to show reference to other reports on ICT use within the area (examples of methodology can be found in many works).

Pls complete the bibliography on the use of VSAT systems e.g.:

M. Sasanuma, H. Uchiyama, T. Nagoya, M. Furukawa, T. Motohisa, Research and development of very small aperture terminals (VSAT) that can be installed by easy operation during disasters – Issues and the solutions for implementing simple and easy installation of VSAT earth station, IEICE Tech. Rep., Vol. 112 (2013), pp. 1–3.

N. Suematsu, H. Oguma, S. Eguchi, S. Kameda, M. Sasanuma, K. Kuroda, Multi-mode SDR VSAT against big disasters, in: European Microwave Conference, EuMC, Nuremberg, 2013. doi: 10.23919/EuMC.2013.6686788.

S. Kameda, T. Okuguchi, S. Eguchi, N. Suematsu, Development of satellite-terrestrial multimode VSAT using software defined radio technology, in: Asia-Pacific Microwave Conference, IEEE, Sendai, 2014.

Response: In response, text capturing literature on the use of digital health to support the delivery of health services in low- and middle-income countries has been added at around line 101. The references the reviewer suggests are all conference proceedings/abstracts that – based on the available metadata – have common authors and appear (although not easily determinable) to cover similar content. Given this, we have included to the most recent suggested article but not the others as a reference.

R1.2.9. Authors write "VSAT remains vulnerable to broader system disruptions" – it would be worthwhile to justify it. There are many resources on the Internet. Examples of literature are here: <https://doi.org/10.15244/pjoes/73907>, <https://doi.org/10.15244/pjoes/73906>, <https://doi.org/10.15244/pjoes/73906>. It would be useful to complete the bibliography in this area as well.

Response: In response, we have added a sentence at around line 238 that reads, "The impact of climatic factors – in particular, rain – on VSAT function are discussed in the literature. [34].”

R1.2.10. There are minor editing errors in the article, such as a period in the wrong place after citations.

Response: The manuscript has been reviewed, and errors addressed.

R1.2.11. Figure 2 is directly taken from the article cited in the bibliography, i.e. Wilk-Jakubowski J (2021) A review on information systems engineering using vsat networks and their development directions. Yugosl J Oper Rres 31 (3): 409–428. doi:10.2298/YJOR200215015W. The Authors did not provide a source for the figure in the caption. If they have the last publisher's approval it should be sent to the publisher. Pls provide a footnote to this caption, i.e. [12] to avoid plagiarism.

Response: Figure 2 has been redrawn, and a footnote added explaining that it is an adaption of Figure 7 in Wilk-Jakubowski's 2021 paper.

**Reviewer #2**

R2.1. [You need to explain that] Communication satellites are physical connections and have a large transmission delay compared to submarine cables.

Response: A sentence reflecting the meaning of this comment has been added at around line 111.

R2.2. The satellite communication with TCP-IP protocol environment, what kind of connection method you are using? for example, DAMA (Demand Assigned Multiple Access) or so on.

Response: While inclusion of this information may provide some context, however, the research is implementation science-oriented with a focus on the impact the introduction of VSAT has on service delivery in Tuvalu, and not on the system's design.

R2.3. How effective is the exchange of data between remote islands and central hospital where the movement of patients is extremely small?

Response: While our study does not set out to measure effectiveness, it has demonstrated the added utility digital connectivity offers. It discusses the cost, time and resource-saving resulting from the VSAT-enabled ability to communicate and exchange data using digital means. In practical terms, these benefits have materialised in enhanced healthcare worker-to-healthcare worker communication regarding patients’ clinical care, examples of telemedicine, and increased opportunities to access training and professional mentoring.

R2.4. In terms of cost, isn't it cheaper to have a patient carry a medium such as a CD-ROM ?

Response: If measured as a per event cost, yes, probably. However, the opportunity VSAT offers go behind data transfer cost savings. As discussed, the digital system has partly addressed barriers previously faced in communication, peer support, collaborative clinical decision-making, data transfer and access to services.

R2.5. There are few references, please add more papers.

Response: Additional references have been added.
